# Biobanking and microRNA – small but with great dividend

**DOI:** 10.1186/1479-5876-10-S2-A50

**Published:** 2012-10-17

**Authors:** Jingfang Ju

**Affiliations:** 1 Translational Research Laboratory, Stony Brook University, School of Medicine, Stony Brook, NY 11794, USA

## 

Biobanking represents a critical resource for personalized translational medicine discovery. It is a great challenge to establish a high quality and comprehensive tissue biobank for cancer research. Many hospitals and clinical centers have large archival existing formalin fixed paraffin embedded (FFPE) tissue depository in the pathology department along with rich history of clinical diagnosis and long term follow up information. This is an existing gold mine for biomarker discovery. Our laboratory has first systematically demonstrated that unlike coding mRNA, a class of non-coding RNA, microRNA (miRNA), is rather stable in FFPE specimens [1]. This discovery established the foundation of miRNA based biomarker discovery using archival FFPE specimens. The superior stability of miRNAs in archival FFPE, and serum samples provides ideal candidates for biomarker discovery using current tissue bank. Mounting evidence showed that post-transcriptional and translational controls mediated by various regulatory molecules, such as RNA binding proteins and miRNAs, are critically important in cancer. Research involved in the translational regulation of suspected genes in cancer has become a new frontier in recent years. Our laboratory was first discovered that a number of miRNAs were regulated by tumor suppressor p53 in colon cancer [2]. Such regulatory mechanism was important in regulating cell proliferation, cycle control and cell death [3]. To investigate the impact of miRNA in chemoresistance to fluoropyrimidines and antifolates, we discovered that miR-215 suppresses the expression of both thymidylate synthase and dihydrofolate reductase [4]. In addition, the expression of miR-215 was directly regulated by p53. The expression of miR-215 was significantly associated with colorectal cancer patient survival [5]. miR-140 modulates chemosensitivity by suppressing HDAC4 expression, and the levels of miR-140 and miR-215 were elevated in colon cancer stem cells [6]. Our recent studies have shown that miR-194 was directly involved in epithelial-to-mesenchymal (EMT) transition, a critical event for tumor progression and metastasis. The expression of BMI-1 protein was suppressed by miR-194 directly at the 3’-UTR region of BMI-1 mRNA [7]. Given the significant role of miRNAs in many aspects of tumor development such as proliferation, cell cycle control, invasion, EMT and maintained tumor stem cell phenotype, we remain hopeful that miRNA based therapeutics, diagnosis and prognosis may emerge in the near future to benefit patients.
